# Effects of reducing sedentary behaviour on heart rate variability and cardio‐metabolic biomarkers in desk workers with untreated high blood pressure

**DOI:** 10.1113/EP093468

**Published:** 2026-04-02

**Authors:** Abdullah Bandar Alansare, Matthew F. Muldoon, Subashan Perera, Kimberly A. Huber, Molly B. Conroy, John M. Jakicic, Bethany Barone Gibbs

**Affiliations:** ^1^ College of Sports Sciences and Physical Activity Department of Exercise Physiology King Saud University Riyadh Saudi Arabia; ^2^ School of Medicine Division of Cardiology Department of Medicine University of Pittsburgh Pittsburgh Pennsylvania USA; ^3^ Departments of Medicine and Biostatistics University of Pittsburgh Pittsburgh Pennsylvania USA; ^4^ Department of Neurology University of Pittsburgh Pittsburgh Pennsylvania USA; ^5^ School of Medicine University of Utah North Salt Lake Utah USA; ^6^ Department of Internal Medicine University of Kansas Medical Center Kansas City Kansas USA; ^7^ School of Public Health West Virginia University Morgantown West Virginia USA

**Keywords:** aldosterone, cardio‐autonomic, glucose, lipids, physical activity, renin

## Abstract

Lower sedentary behaviour (SB) has been associated with reduced cardiovascular disease and mortality. Yet, few trials have evaluated cardiovascular mechanisms of benefit. We examined the effects of a SB reduction intervention on heart rate variability (HRV) and cardio‐metabolic biomarkers. We evaluated secondary outcomes in RESET‐BP, a randomized clinical trial testing the effects of a 3‐month SB reduction intervention on blood pressure (BP) in desk workers with untreated high BP. The intervention included health coaching, sit–stand desks, wearable activity prompters and text messages that reduced SB by increasing standing (∼1 h/day) and stepping (∼5 min/day). Resting HRV was assessed in a subset of participants. Fasting blood plasma and serum were drawn to evaluate several cardio‐metabolic biomarkers, including glucose, insulin, lipids, aldosterone and plasma renin activity. Analysis of covariance models examined the effect of the intervention on outcomes. Participants with baseline and follow‐up measures of HRV (*n* = 146) and cardio‐metabolic biomarkers (*n* = 188) were included. No significant intervention effects were observed for any measures of HRV, glucose, insulin or lipids (all *P* > 0.05). Three‐month changes in aldosterone (difference = −1.5 ng/dL; *P* = 0.0370) and plasma renin activity (difference = −0.2 ng/mL/h; *P* = 0.0462) were significantly lower in the intervention compared to the control condition at the 3‐month follow‐up. Reducing SB with mostly standing did not improve HRV or cardio‐metabolic biomarkers. However, the observed intervention effects on plasma renin and aldosterone encourage future research to confirm that SB reduction suppresses the renin–angiotensin–aldosterone system activity.

## Abstract (highlights)

- **What is the central question of this study?**
  Does a sedentary behaviour reduction intervention improve heart rate variability and cardio‐metabolic biomarkers in desk workers with untreated, high blood pressure?
- **What is the main finding and its importance?**
  Short‐term sedentary behaviour reduction suppressed plasma renin activity and aldosterone in desk workers with untreated, high blood pressure. However, it did not improve heart rate variability, fasting glucose, insulin or lipids. This study provides new insights regarding the mechanism through which sedentary behaviour reduction may improve cardiovascular health. Sedentary behaviour reduction may be a non‐pharmacological approach to modulate renin–angiotensin–aldosterone system activity.

## 1 INTRODUCTION

The global prevalence of cardiovascular disease (CVD) is projected to increase by 90% by 2050, with 35.6 million people worldwide forecasted to die from CVD in 2050 (Chong et al., 2025). These alarming numbers have compelled the scientific community and decision‐makers to establish clinical recommendations to prevent and manage CVD (Arnett et al., 2019; Parikh et al., 2021). Lifestyle modifications, such as accumulating recommended levels of moderate‐to‐vigorous intensity physical activity (MVPA), are a frontline strategy for CVD prevention and management (Arnett et al., 2019; Parikh et al., 2021). Nonetheless, half of US adults do not meet physical activity (PA) recommendations and thus do not realize the associated health benefits (Abernethy et al., 2025). Alternative and practical lifestyle modification strategies are needed to prevent and manage CVD.

Separate from insufficient MVPA, sedentary behaviour (SB) is prevalent (Matthews et al., 2021) and increasingly recognized in observational studies as a potential modifiable lifestyle factor that increases the risk of CVD (Katzmarzyk et al., 2019). SB is defined as waking time spent in seated, lying, or reclining postures with minimal energy expenditure (Tremblay et al., 2017). Though the pathways through which SB leads to CVD are still under investigation, cardio‐autonomic and cardio‐metabolic impairments are proposed mechanisms (Pinto et al., 2023). Higher time spent in SB, especially if uninterrupted, may reduce muscular activity and promote extremity blood pooling, causing a decrease in venous return and blood pressure (BP) (Pinto et al., 2023). Consequently, vagal activity could be suppressed, resulting in augmented sympathetic outflow and activation of the renin–angiotensin–aldosterone system (RAAS) activity (Mueller, 2008; Pinto et al., 2023). Concurrently, reduced muscle activity with high levels of SB would reduce metabolic activity, which could impair the regulation of glucose, insulin and lipid (Pinto et al., 2023). Over time, these cardio‐autonomic and cardio‐metabolic alterations could become chronic maladaptations, leading to CVD (Mueller, 2008; Pinto et al., 2023).

Notably, robust evidence demonstrates that interventions which increase MVPA can improve the cardio‐autonomic (i.e., vagal activity) and cardio‐metabolic (i.e., glucose, insulin and lipids) functions (Lin et al., 2015; Grässler et al., 2021). Nonetheless, whether these same benefits can be realized from reducing SB and substituting it with light intensity PA (e.g., standing or activities of daily living) has not been established in randomized trials. A greater understanding of whether and how reducing SB influences such specific aspects of cardiovascular health is needed to inform targeted prevention and treatment approaches.

The Reducing Sedentary Behavior on Blood Pressure (RESET‐BP) Study was a randomized clinical trial that examined the effects of a 3‐month SB reduction intervention on BP in desk workers with untreated high BP. As previously reported (Gibbs et al., 2024), the trial was successful at decreasing SB and increasing standing and stepping, yet no differences in BP (systolic BP −0.22 mmHg, *P* = 0.808; diastolic BP 0.13 mmHg, *P* = 0.827) were observed. RESET‐BP also measured secondary outcomes in a subset of participants, including vagal activity via heart rate variability (HRV) assessment and cardio‐metabolic biomarkers (i.e., glucose, insulin, lipids, aldosterone and plasma renin). Previous research observed HRV or cardio‐metabolic improvements following PA interventions without changes in BP (Molina et al., 2011; Nygaard et al., 2017; Papp et al., 2013). As such, although the current SB reduction intervention did not influence BP, whether it improved HRV or cardio‐metabolic biomarkers remains a valuable research question. Thus, this study examined the effects of the RESET‐BP 3‐month SB reduction intervention on these secondary outcomes (i.e., HRV, glucose, insulin, lipids, aldosterone and plasma renin) in desk workers with elevated, untreated BP. We hypothesized that the SB reduction intervention would improve HRV and these cardio‐metabolic biomarkers compared to the control condition.

## 2 METHODS

### 2.1 Ethical approval

The report of this study was completed following the Consolidated Standards of Reporting Trials (CONSORT) guidelines (Moher et al., 2001). The study was registered on clinicaltrials.gov (NCT03307343). The study conformed to the standards set by the last revision of the *Declaration of Helsinki*. All research procedures were approved by the University of Pittsburgh's Human Research Protections Office (STUDY19030297), and all participants provided a written informed consent.

### 2.2 Participants and experimental design

The RESET‐BP randomized controlled trial recruited desk workers between January 2018 and August 2022 in the Pittsburgh Metropolitan Area, PA, USA. The detailed protocol (Gibbs et al., 2021) and primary outcomes (Gibbs et al., 2024) are reported elsewhere. Briefly, the inclusion criteria for the RESET‐BP study were: (1) aged 21–65 years, (2) systolic BP 120–159 mmHg or diastolic BP 80–99 mmHg, (3) reporting less than 150 min/week of MVPA, and (4) currently engaging in deskwork for ≥20 h/week. Individuals were excluded if they: (1) were using medications to treat elevated BP, (2) were using glucose‐controlling medications, (3) had resting systolic BP ≥160 mmHg or diastolic BP ≥100 mmHg, or (4) had a history of CVD (e.g., stroke, chronic heart failure) or any other medical condition (e.g., musculoskeletal disorders, ongoing chemotherapy) that could affect their ability to safely participate in the intervention. Eligible participants were randomized into one of the two arms of the parallel randomized controlled trial, either a 3‐month SB reduction intervention or a no‐contact control condition (Gibbs et al., 2021). Cardio‐autonomic and cardio‐metabolic biomarker assessments were sequentially obtained at baseline (within 30 days of randomization) and 3‐month follow‐up (91 to 101 days after randomization) by blinded assessors.

To be included in the current analyses, participants had to have completed baseline and follow‐up assessments of cardio‐autonomic or cardio‐metabolic biomarkers. Of the 271 participants who enrolled in the RESET‐BP study, 263 (97%) completed follow‐up assessment of the primary outcome (resting BP). Both secondary outcomes were missed in a portion of participants at baseline, follow‐up, or both due to primary outcome‐only assessments during the interval from March–July of 2020, when assessments were restricted due to COVID‐19. Also, cardio‐autonomic assessments were added mid‐way through the enrolment period, resulting in only 146 with complete baseline and follow‐up data on cardio‐autonomic outcomes. Cardio‐metabolic biomarkers were further limited if either baseline or follow‐up sampling was refused or unsuitable (e.g., haemolysed, clotted or insufficient volume), resulting in a final sample size of 188 with both time points (Gibbs et al., 2021).

### 2.3 Randomization

Participants were randomized in a 1:1 ratio into the intervention or control condition, with random block sizes of 4–6, stratified by sex (male or female) and BP stage (elevated, stage one or stage two) (Gibbs et al., 2021).

### 2.4 Intervention

A multicomponent intervention strategy was implemented to reduce SB, especially prolonged bouts, by increasing standing and stepping (Gibbs et al., 2021). The intervention consisted of health coaching sessions twice a month, provision of a height‐adjustable workstation, a wearable activity prompter (i.e., Fitbit Flex 2) and phone texts. Each hour while working, participants were encouraged to stand for 15–30 min and take a movement break. Participants were counselled to generally sit less and move more during non‐working hours. As reported previously (Gibbs et al., 2024), the rates of adherence to the intervention were high throughout the 3‐month intervention period, with >95% of intervention lessons delivered, >94% wearing their Fitbit, and >83% reporting self‐monitoring standing and movement breaks.

### 2.5 Measurements

#### 2.5.1 General characteristics, anthropometrics and blood pressure

Participant age, sex, ethnicity and race were self‐reported using a standardized demographic questionnaire. At baseline, two consecutive body height (cm) and weight (kg) readings were taken utilizing a wall‐mounted stadiometer (Perspective Enterprises, Portage, MI, USA) and a digital scale (WB‐110A; Tanita, Tokyo, Japan), respectively. The average of the readings was then used to compute body mass index (BMI) (kg/m^2^). As previously reported in detail, the primary outcomes of office blood pressure (HEM‐907, OMRON Healthcare, Kyoto, Japan) and 24‐h ambulatory blood pressure (Oscar II, SunTech Medical, Morrisville, NC, USA) were measured by a standardized protocol at baseline and follow‐up (Gibbs et al., 2024).

#### 2.5.2 Sedentary behaviour and physical activity

Participants wore two activity monitors at baseline and follow‐up for 7 days and completed a concurrent wear diary reporting working times and sleep/non‐wear periods (for additional details, see previous report; Gibbs et al., 2021). With sufficient valid wear days (≥4 days with ≥10 h/day) (Matthews et al., 2012), data were processed to provide device‐based assessment of sedentary, standing, and stepping times (thigh‐worn activPAL, PalTechnologies, Glasgow, UK) (Edwardson et al., 2017) and MVPA (waist‐worn ActiGraph GT3X, ActiGraph LLC, Pensacola, FL, USA) (Sasaki et al., 2011). Processing included using device‐specific software and semi‐automated data cleaning to segregate work from non‐work intervals and to remove non‐wear and sleep periods. Daily durations of each behaviour were calculated and then averaged over valid days to provide daily estimates of sedentary, standing and stepping time (intervention targets) as well as weekly estimates of MVPA (not targeted by the intervention). Herein, baseline, follow‐up, changes and group comparisons are provided for the subsamples included in this report.

#### 2.5.3 Heart rate variability

HRV, the fluctuations in periods between consecutive heartbeats, was measured from each participant to analyse and compare baseline and follow‐up assessments (Task Force of the European Society of Cardiology and the North American Society of Pacing and Electrophysiology, 1996). Supine HRV was measured continuously for 10 min utilizing a validated instrument to monitor R‐R intervals (Polar V800 monitor with Polar H10 heart rate strap; Polar Electro, Inc., Kempele, Finland) (Giles et al., 2016), with the first 5 min considered the rest period and the final 5 min used for analysis. The male participants wore the heart rate strap on their chest, below the pectoralis muscle, with the middle of the strap placed at the sternum. For female participants, the heart rate strap was secured around their chest, at the level of the bra band, beneath the breast tissue. During the entire measurement period, room lights were turned off, and participants were instructed to breathe normally and not to move, talk or sleep.

The collected R‐R interval data were then processed using Kubios Premium HRV analysis software (version 3.3.1, MATLAB, MathWorks, Inc., Natick, MA, USA). To minimize the impacts of postural transitioning on HRV, the first 5 min of the R‐R interval data was discarded, and the subsequent 5 min was examined for acceptability using the existing guidelines (Laborde et al., 2017). Artifacts were identified by implementing the automatic correction. Files exceeding 5% artifacts were discarded, and those with ≤5% were visually inspected for any further distortion or missing R waves. The validated files were then used to extract the following HRV indices: the standard deviation of normal R‐R intervals (SDNN), the root mean square of the successive differences (RMSSD), low‐frequency power (LF), high‐frequency power (HF) and LF/HF (Laborde et al., 2017).

#### 2.5.4 Cardio‐metabolic biomarkers

Blood plasma and serum were obtained from upright seated posture from each participant to analyse and compare baseline and follow‐up cardio‐metabolic biomarkers, including fasting glucose and insulin, insulin resistance (Homeostatic Model Assessment for Insulin Resistance [HOMA‐IR]), total cholesterol, low‐density lipoprotein (LDL), high‐density lipoprotein (HDL), triglycerides, aldosterone and renin. Blood samples were drawn after an overnight fast and at least 30 min of continuous sitting to reduce any influence of PA or postural changes. All samples were scheduled to be obtained in the morning (before noon), though a few samples were collected slightly after noon to accommodate participant schedules. Blood samples were processed using a centrifuge, and 2 µL aliquots were stored in cryovials at −80°F (−62.22°C) until the end of the study to reduce batch effects of assays. Standard laboratory methods were used to measure total cholesterol, HDL, triglycerides, and fasting glucose and insulin. LDL was calculated as total cholesterol − HDL − (triglycerides/5). HOMA‐IR was calculated as fasting insulin × fasting glucose/405. ELISA was used to measure plasma renin activity (MyBiosource, Vancouver, British Columbia, Canada; inter‐assay coefficients of variation on controls between 8% and 19%) and plasma aldosterone (Abnova, Taipei, Taiwan; inter‐assay coefficients of variation on controls between 6% and 11%). Aldosterone‐to‐renin ratio was calculated as aldosterone/plasma renin activity.

### 2.6 Statistical analyses

The characteristics of the included participants were described using summary statistics and compared across randomized groups using independent Student's *t*‐test or chi‐square test, as appropriate. Those excluded were also compared to those included using similar analyses. The effects of the intervention on behaviour durations, HRV and cardio‐metabolic biomarkers were estimated using analysis of covariance models with the baseline to 3‐month change in outcomes as the dependent variable, the baseline value of the outcome as a covariate, and the intervention arm as the effect of interest. HRV variables were analysed after being natural log‐transformed (ln). In addition, the analyses of plasma renin activity and aldosterone were repeated after being log_10_ transformed to evaluate the robustness of findings. Finally, partial correlations between changes in plasma renin activity and aldosterone with changes in blood pressure, adjusting for baseline values, were calculated overall and by randomized group. The significance level was set as *P* < 0.05. All analyses were performed using SAS software version 9.4 (SAS Institute, Inc., Cary, NC, USA).

## 3 RESULTS

Table 1 displays baseline characteristics of the subsamples of participants who were included in the HRV (*n* = 146) and cardio‐metabolic (*n* = 188) biomarkers analyses, by randomized group. Participants included in the HRV analyses were comparable between randomized groups (*P* > 0.05 for all), except for age (intervention: 46.5 vs. control: 41.9 years; *P* < 0.0108). The participants included in the cardio‐metabolic biomarkers analyses were similar regarding all characteristics between the randomized groups.

**TABLE 1:** Participant characteristics in subsamples by randomized group.

Variable	HRV subsample (*n* = 146)			Cardio‐metabolic biomarkers subsample (*n* = 188)		
	Intervention (*n* = 76) (mean ± SD or *n* (%))	Control condition (*n* = 70) (mean ± SD or *n* (%))	*P*	Intervention (*n* = 93) (mean ± SD or *n* (%))	Control condition (*n* = 95) (mean ± SD or *n* (%))	*P*
Age (years)	**46.5 ± 10.4**	**41.9 ± 10.9**	**0.0108**	47.4 ± 11.6	44.3 ± 11.7	0.0746
Sex						
Female Male	38 (50.0) 38 (50.0)	36 (51.4) 34 (48.6)	0.8631	53 (55.8) 42 (44.2)	53 (57.0) 40 (43.0)	0.8683
Ethnicity						
Non‐Hispanic Hispanic Not reported	72 (94.4) 4 (5.3) 0 (0.0)	68 (97.1) 1 (1.4) 1 (1.4)	0.2796	90 (94.7) 5 (5.3) 0 (0.0)	89 (95.7) 4 (4.3) 0 (0.0)	1.0000
Race						
White Black Pacific Islander Asian Other Multiracial	64 (84.2) 6 (7.9) 0 (0.0) 2 (2.6) 2 (2.6) 2 (2.6)	62 (88.6) 4 (5.7) 1 (1.4) 1 (1.4) 2 (2.9) 0 (0.0)	0.7400	81 (85.3) 5 (5.3) 0 (0.0) 5 (5.3) 1 (1.1) 3 (3.2)	83 (89.3) 5 (5.4) 1 (1.1) 3 (3.2) 1 (1.1) 0 (0.0)	0.5001
Resting BP (mmHg)						
Systolic Diastolic	129.5 ± 8.3 82.8 ± 7.1	126.9 ± 8.9 83.4 ± 6.3	0.0714 0.5773	129.4 ± 8.5 82.5 ± 7.4	129.3 ± 9.7 82.9 ± 6.2	0.9398 0.7149
BMI (kg/m^2^)	30.4 ± 5.4	32.3 ± 7.4	0.0788	30.4 ± 6.1	30.1 ± 6.4	0.8044

*Note*: Bold indicates significant difference (*P* < 0.05). Abbreviations: BMI, body mass index; BP; blood pressure.

Supporting information Table S1 compares baseline characteristics between those included versus excluded from the analyses due to missing HRV or blood measures. Only being female or from a racial minority was more common in those excluded from the HRV analyses, and only BMI was higher in those excluded from the cardio‐metabolic biomarkers analyses.

Table 2 displays baseline, follow‐up and differences in device‐based measures of sedentary time, standing time, stepping time and MVPA in the two subsamples. Similar to the published results in the full sample (Gibbs et al., 2024), the intervention significantly reduced SB (adjusted mean differences ranged between −1.2 and −1.5 h/day) and increased standing (adjusted mean differences ranged between 0.9 to 1.3 h/day) compared with the control condition (*P* < 0.05 for all) in both subsamples. Although the changes were small and comparable across the two subsamples, the increase in stepping among the intervention compared to the control condition was only significant in the cardio‐metabolic biomarker subsample (adjusted mean differences were 0.1 h/day; *P* < 0.05). Notably, all significant changes observed in these activities were mainly attributed to changes in workdays. Also, as intended by the RESET‐BP intervention, no difference was observed in MVPA between groups.

**TABLE 2:** Changes in SB and PA comparing the intervention versus control condition for the HRV subsample (*n* = 146) and cardio‐metabolic biomarker subsample (*n* = 188).

	Intervention			Control condition				
	Baseline (mean ± SD)	Follow‐up (mean ± SD)	Change (mean ± SD)	Baseline (mean ± SD)	Follow‐up (mean ± SD)	Change (mean ± SD)	Adjusted difference (mean ± SE)	*P*
HRV subsample (*n* = 146)								
*n*	76			70				
SB (h/day)								
Total Working time Non‐working time	**10.9 ± 1.3** **6.7 ± 1.3** 6.2 ± 1.3	**9.7 ± 1.6** **5.1 ± 1.6** 6.1 ± 1.3	**−1.1 ± 1.4** **−1.6 ± 1.7** −0.1 ± 1.4	**11.1 ± 1.6** **6.8 ± 1.5** 6.2 ± 1.6	**11.1 ± 1.8** **6.7 ± 1.9** 6.4 ± 1.5	**−0.0 ± 1.4** **−0.1 ± 1.8** 0.2 ± 1.5	**−1.2 ± 0.2** **−1.5 ± 0.3** −0.3 ± 0.2	**<0.0001** **<0.0001** 0.1273
Standing time (h/day)								
Total Working time Non‐working time	**3.5 ± 1.1** **1.4 ± 1.1** 2.5 ± 1.0	**4.5 ± 1.5** **2.8 ± 1.5** 2.6 ± 1.0	**1.0 ± 1.2** **1.4 ± 1.5** 0.1 ± 0.8	**3.2 ± 1.4** **1.2 ± 1.0** 2.4 ± 1.1	**3.4 ± 1.5** **1.4 ± 1.1** 2.4 ± 1.1	**0.2 ± 1.2** **0.2 ± 1.1** 0.0 ± 0.9	**0.9 ± 0.2** **1.3 ± 0.2** 0.1 ± 0.1	**<0.0001** **<0.0001** 0.3953
Stepping time (h/day)								
Total Working time Non‐working time	1.6 ± 0.5 0.6 ± 0.3 1.1 ± 0.4	1.6 ± 0.5 0.7 ± 0.3 1.1 ± 0.4	0.1 ± 0.4 0.1 ± 0.3 0.0 ± 0.4	1.5 ± 0.7 0.6 ± 0.4 1.1 ± 0.5	1.5 ± 0.7 0.6 ± 0.5 1.1 ± 0.5	0.06 ± 0.5 0.1 ± 0.3 −0.0 ± 0.5	0.0 ± 0.1 0.1 ± 0.0 0.1 ± 0.1	0.5287 0.1016 0.4441
MVPA (min/week)	256.8 ± 136.5	248.4 ± 144.2	−8.4 ± 107.8	268.8 ± 164.8	277.4 ± 180.0	9.3 ± 138.6	−21.5 ± 19.8	0.2801
Cardio‐metabolic subsample (*n* = 188)								
*n*	93			95				
SB (h/day)								
Total Working time Non‐working time	**11.3 ± 1.4** **6.8 ± 1.3** 6.6 ± 1.5	**10.0 ± 1.6** **5.3 ± 1.7** 6.3 ± 1.4	**−1.3 ± 1.4** **−1.6 ± 1.7** −0.3 ± 1.4	**10.9 ± 1.5** **6.5 ± 1.3** 6.4 ± 1.5	**11.0 ± 1.7** **6.3 ± 1.6** 6.4 ± 1.5	**0.0 ± 1.5** **−0.2 ± 1.5** −0.0 ± 1.3	**−1.2 ± 0.2** **−1.2 ± 0.2** −0.2 ± 0.2	**<0.0001** **<0.0001** 0.2066
Standing time (h/day)								
Total Working time Non‐working time	**3.4 ± 1.1** **1.4 ± 1.0** 2.6 ± 1.0	**4.5 ± 1.5** **2.7 ± 1.3** 2.6 ± 1.0	**1.1 ± 1.2** **1.4 ± 1.4** 0.0 ± 0.9	**3.5 ± 1.3** **1.2 ± 0.8** 2.6 ± 1.1	**3.6 ± 1.4** **1.4 ± 1.1** 2.5 ± 1.1	**0.1 ± 1.2** **0.2 ± 1.0** −0.1 ± 0.9	**1.0 ± 0.2** **1.2 ± 0.2** 0.07 ± 0.1	**<0.0001** **<0.0001** 0.5293
Stepping time (h/day)								
Total Working time Non‐working time	**1.5 ± 0.5** **0.6 ± 0.3** 1.1 ± 0.5	**1.6 ± 0.5** **0.7 ± 0.3** 1.1 ± 0.4	**0.12 ± 0.4** **0.12 ± 0.2** 0.02 ± 0.4	**1.5 ± 0.6** **0.6 ± 0.4** 1.1 ± 0.5	**1.54 ± 0.6** **0.61 ± 0.4** 1.07 ± 0.5	**−0.0 ± 0.4** **0.0 ± 0.2** −0.1 ± 0.4	**0.1 ± 0.1** **0.1 ± 0.0** 0.1 ± 0.1	**0.0299** **0.0077** 0.1298
MVPA (min/week)	257.9 ± 148.4	266.6 ± 149.6	13.4 ± 108.8	269.0 ± 158.0	277.0 ± 159.1	7.7 ± 117.7	2.1 ± 16.1	0.8962

*Note*: Bold indicates significant difference (*P* < 0.05). Abbreviations: MVPA, moderate to vigorous physical activity; PA, physical activity; SB, sedentary behaviour.

Table 3 displays baseline, follow‐up and differences in HRV. No significant differences were observed between groups for any HRV measures (*P* > 0.05 for all). Because higher HRV reflects healthier cardio‐autonomic regulation, the non‐significant differences observed were typically negligible (approximately 0) or favoured the control condition. A sensitivity analysis including age as an additional covariate due to the baseline imbalance did not materially affect the results.

**TABLE 3:** Changes in HRV comparing the intervention versus control condition (*n* = 146).

	Intervention (*n* = 76)			Control condition (*n* = 70)			Adjusted difference (mean ± SE)	*P*
	Baseline (mean ± SD)	Follow‐up (mean ± SD)	Change (mean ± SD)	Baseline (mean ± SD)	Follow‐up (mean ± SD)	Change (mean ± SD)		
ln SDNN (ms)	3.3 ± 0.5	3.4 ± 0.5	0.0 ± 0.3	3.4 ± 0.5	3.5 ± 0.6	0.1 ± 0.4	−0.0 ± 0.1	0.4341
ln RMSSD (ms)	3.3 ± 0.6	3.3 ± 0.6	0.0 ± 0.4	3.3 ± 0.6	3.4 ± 0.7	0.1 ± 0.5	−0.1 ± 0.1	0.3519
ln LF (ms^2^)	5.8 ± 1.0	6.0 ± 1.0	0.2 ± 0.9	6.1 ± 1.0	6.1 ± 1.4	0.1 ± 1.1	0.0 ± 0.2	0.8914
ln HF (ms^2^)	5.5 ± 1.2	5.6 ± 1.3	0.1 ± 0.8	5.6 ± 1.3	5.8 ± 1.5	0.1 ± 1.0	−0.0 ± 0.1	0.7872
ln LF/HF ratio	2.1 ± 2.4	2.2 ± 2.6	0.2 ± 2.9	2.3 ± 2.5	2.5 ± 3.8	0.2 ± 3.6	−0.2 ± 0.5	0.7438

Abbreviations: HF, high‐frequency power; LF, low‐power frequency; ln, natural log‐transformed; RMSSD, root mean square of the successive differences; SDNN, standard deviation of normal R‐R intervals.

Table 4 displays baseline, follow‐up and differences in cardio‐metabolic biomarkers. No significant differences were observed between groups for fasting glucose, insulin, HOMA‐IR or lipid measures (*P* > 0.05 for all). The intervention group had a decline in aldosterone (adjusted difference = −1.5 ng/dL; *P* = 0.0370) and plasma renin activity (adjusted difference = −0.2 ng/mL/h; *P* = 0.0462) at the 3‐month follow‐up relative to the control condition. The findings were similar after log_10_ transformation. Of note, inspection of the aldosterone values indicated the effect was driven primarily by a rise in aldosterone in the control participants. The intervention did not significantly change the aldosterone/renin ratio compared to the control.

**TABLE 4:** Changes in cardio‐metabolic biomarkers comparing the intervention versus control condition (*n* = 188).

	Intervention (*n* = 93)			Control condition (*n* = 95)			Adjusted difference (mean ± SE)	*P*
	Baseline (mean ± SD)	Follow‐up (mean ± SD)	Change (mean ± SD)	Baseline (mean ± SD)	Follow‐up (mean ± SD)	Change (mean ± SD)		
Glucose (mg/dL)	98.0 ± 33.1	95.1 ± 28.8	−2.8 ± 13.8	91.9 ± 15.3	92.6 ± 11.7	0.8 ± 12.0	−2.0 ± 1.6	0.2252
Insulin (IU)	15.9 ± 16.7	14.5 ± 9.1	−1.4 ± 15.4	12.8 ± 10.1	12.7 ± 11.3	−0.0 ± 6.1	0.4 ± 1.3	0.7233
HOMA‐IR	4.1 ± 5.3	3.5 ± 2.6	−0.6 ± 4.7	3.0 ± 2.9	3.1 ± 4.0	0.1 ± 2.1	−0.1 ± 0.4	0.8078
Total cholesterol (mg/dL)	194.8 ± 32.3	192.5 ± 38.9	−2.3 ± 26.9	196.2 ± 39.1	194.6 ± 38.5	−1.6 ± 29.6	−1.0 ± 4.0	0.7926
LDL cholesterol (mg/dL)	115.5 ± 30.0	112.6 ± 32.3	−3.3 ± 16.7	114.7 ± 33.1	114.0 ± 33.2	−0.6 ± 25.3	−2.4 ± 3.1	0.4427
HDL cholesterol (mg/dL)	54.3 ± 15.4	54.1 ± 15.3	−0.2 ± 5.0	59.1 ± 19.4	58.8 ± 17.6	−0.3 ± 11.2	−0.7 ± 1.2	0.5431
Triglycerides (mg/dL)	131.4 ± 84.0	125.2 ± 80.7	−6.2 ± 68.8	112.2 ± 57.1	108.7 ± 54.6	−3.5 ± 33.7	3.5 ± 7.2	0.6270
Aldosterone (ng/dL)	**9.5 ± 8.5**	**9.3 ± 8.0**	**−0.1 ± 4.9**	**11.1 ± 11.5**	**11.9 ± 11.8**	**1.3 ± 4.6**	**−1.5 ± 0.7**	**0.0370**
Log_10_ aldosterone (ng/dL)	**0.89 ± 0.26**	**0.87 ± 0.28**	**−0.01 ± 0.22**	**0.91 ± 0.32**	**0.95 ± 0.31**	**0.06 ± 0.20**	**−0.07 ± 0.03**	**0.0266**
Plasma renin activity (ng/mL/h)	**1.4 ± 1.5**	**1.2 ± 1.3**	**−0.2 ± 1.0**	**1.4 ± 1.2**	**1.5 ± 1.1**	**0.1 ± 0.8**	**−0.2 ± 0.1**	**0.0462**
Log_10_ plasma renin activity (ng/mL/h)	**−0.06 ± 0.42**	**−0.11 ± 0.42**	**−0.06 ± 0.31**	**0.01 ± 0.39**	**0.06 ± 0.33**	**0.05 ± 0.26**	**−0.12 ± 0.04**	**0.0021**
Aldosterone/renin ratio	14.9 ± 37.9	15.4 ± 26.4	1.2 ± 26.5	13.2 ± 18.6	13.1 ± 26.9	0.5 ± 19.4	1.1 ± 3.0	0.7081

*Note*: Bold indicates significant difference (*P* < 0.05). Abbreviations: HDL, high‐density lipoprotein; LDL, low‐density lipoprotein.

Table 5 displays partial correlations between changes in plasma renin activity, aldosterone and their ratio with changes in office blood pressure and 24‐h blood pressure. In the full sample, as expected, direct correlations (*r* ranged from 0.15 to 0.40; *P* < 0.05) were observed between changes in the aldosterone/renin ratio and changes in office systolic and diastolic blood pressure and 24‐h systolic blood pressure. Other associations were not statistically significant. When stratified by randomized group, the only correlation that remained statistically significant was a direct association between changes in aldosterone/plasma renin ratio and office systolic blood pressure in the control group (*r* = 0.47; *P* < 0.001).

**TABLE 5:** Partial correlations between changes in blood pressure and changes in RAAS biomarkers adjusting for baseline values.

	*r* (*P*‐value)		
Variable	Change in plasma renin activity (ng/mL/h)	Change in aldosterone (ng/dL)	Change in aldosterone/renin ratio
All participants (*n* = 188)			
Change in office SBP (mmHg)	−0.02 (0.841)	0.04 (0.644)	**0.40 (<0.001)**
Change in office DBP (mmHg)	0.02 (0.830)	0.00 (0.990)	**0.15 (0.046)**
Change in 24‐h SBP (mmHg)	−0.08 (0.323)	−0.04 (0.610)	**0.16 (0.033)**
Change in 24‐h DBP (mmHg)	−0.02 (0.800)	−0.03 (0.742)	0.14 (0.076)
Intervention (*n* = 93)			
Change in office SBP (mmHg)	0.11 (0.338)	0.05 (0.675)	0.18 (0.095)
Change in office DBP (mmHg)	0.10 (0.385)	0.05 (0.671)	0.10 (0.357)
Change in 24‐h SBP (mmHg)	−0.08 (0.474)	0.01 (0.893)	0.15 (0.181)
Change in 24‐h DBP (mmHg)	−0.03 (0.806)	−0.03 (0.810)	0.09 (0.410)
Control condition (*n* = 95)			
Change in office SBP (mmHg)	−0.14 (0.201)	0.01 (0.910)	**0.47 (<0.001)**
Change in office DBP (mmHg)	−0.02 (0.878)	0.013 (0.906)	0.14 (0.196)
Change in 24‐h SBP (mmHg)	−0.10 (0.379)	−0.13 (0.251)	0.18 (0.101)
Change in 24‐h DBP (mmHg)	0.02 (0.886)	0.00 (0.966)	0.21 (0.060)

*Note*: Bold indicates significant correlations (*P* < 0.05). Abbreviations: DBP, diastolic blood pressure; *r*, Pearson's correlation coefficient; RAAS, renin–angiotensin–aldosterone system; SBP, systolic blood pressure.

## 4 DISCUSSION

The present report is the first, to our knowledge, to examine the effects of a SB reduction intervention on HRV and cardio‐metabolic biomarkers in desk workers with untreated high BP. Despite notable decreases in sedentary time and increases in standing time of about 7 h/week, the intervention did not affect HRV, fasting glucose, insulin, HOMA‐IR or lipids. Yet, the intervention group had significantly lower aldosterone and plasma renin activity compared to the control condition at the 3‐month follow‐up. These findings suggest that the SB reduction intervention modestly suppressed the RAAS activity. Although impairments in vagal activity, glucose, insulin, or lipids do not seem to be important mechanisms that link SB to CVD, compromised RAAS activity is a possible underlying mechanism. This biological signal may provide insight into how SB reduction interventions that mostly increase standing could promote cardiovascular health.

### 4.1 Effects on HRV

Research examining SB and HRV is limited. Though a handful of observational studies mostly find no associations between SB and HRV, they are typically cross‐sectional and limited by self‐report measures of SB (Alansare et al., 2021). Considering device‐based SB within specific domains (i.e., occupational and non‐occupational), the Dutch NOMAD Study of 126 working adults found that occupational SB was associated with reduced (worse) night‐time HRV, while non‐occupational SB was not related to HRV across a variety of occupations (Hallman et al., 2015). In contrast, our cross‐sectional analysis of baseline data of the RESET‐BP study found that occupational SB was correlated with higher HRV, whereas non‐occupational SB was associated with lower HRV in desk workers (Alansare, Paley et al., 2023). Furthermore, our recent cross‐sectional analysis in 286 working and non‐working women found that isotemporal substitution of device‐measured SB with MVPA accumulated in bouts lasting at least 10 min was associated with more favourable HRV (Alansare, Gibbs et al., 2023); yet, substitution of SB with light intensity PA was not associated with any benefit. This final study is consistent with the null findings in this report, since the RESET‐BP intervention reduced SB and increased light‐intensity activity with no change in MVPA.

We are aware of only one other longitudinal study in 53 patients with mild hypertension or stable angina enrolled in a 6‐month lifestyle education programme (Nakayama et al., 2021). Participants were separated *post hoc* based on whether they decreased or increased device‐measured SB, and the HF index measured during sleep was significantly higher among those who decreased SB. However, this inference is limited by the small sample and possible selection bias due to the comparison groups being established *post hoc*. Results from RESET‐BP, which found no significant effects of a 3‐month SB reduction intervention on SDNN, RMSSD, LF, HF or LF/HF in desk workers with elevated, untreated BP, are more robust given the rigorous randomized trial design with a control condition and larger sample size. Taken together, most observational data and our experimental trial suggest that reducing SB primarily by increasing standing during desk work does not appear to improve HRV. Thus, impaired vagal activity does not seem to be a significant mechanism through which SB influences CVD risk.

### 4.2 The effects on cardio‐metabolic biomarkers

The SB reduction intervention in our study did not affect several cardio‐metabolic biomarkers, including fasting glucose, insulin, HOMA‐IR, LDL, HDL, total cholesterol or triglycerides. Our findings are comparable to those from a recent meta‐analysis of 33 prior trials with interventions to reduce SB. While the meta‐analysis differed in finding small, beneficial effects on HDL (+0.7 mg/dL, *P* < 0.001) and insulin (−0.2 IU, *P* = 0.047), findings were similar to ours with non‐significant pooled reductions in glucose, LDL, total cholesterol and triglycerides (Hadgraft et al., 2021). Baseline characteristics, intervention durations and components, magnitude of SB reduction, and sample size may explain the inconsistent findings for HDL and insulin, a notion supported by the large heterogeneity noted by the authors performing the meta‐analysis (Hadgraft et al., 2021). Collectively, SB reduction interventions appear to have small or no benefits to several cardio‐metabolic risk factors, suggesting that several cardio‐metabolic impairments may not be crucial mechanisms that link between SB and CVD.

Interestingly, the present study revealed that the SB reduction intervention significantly reduced RAAS‐specific cardio‐metabolic biomarkers, including aldosterone and plasma renin activity. This suggests suppressed RAAS activity in the intervention group compared to the control condition, a potential cardiovascular benefit of SB reduction. Further, changes in the aldosterone/renin ratio correlated with changes in blood pressure in the control participants but not in those assigned to the SB intervention. RAAS activity plays multiple roles in cardiovascular health and disease, such that heightened RAAS activity across a prolonged duration leads to CVD while pharmacological inhibition of the RAAS reduces CVD (Patel et al., 2017; Zhao et al., 2025). Unhealthy lifestyle behaviours, such as low levels of PA, could activate the RAAS, potentially through impaired baroreflex‐mediated sympathoexcitation (Mueller, 2008). A high aldosterone/renin ratio is often found to predict hypertension (Arnold et al., 2023), but it was unaffected by our SB intervention. As we are unaware of other studies evaluating these outcomes, further investigation is warranted, including into the clinical meaningfulness of these small differences in plasma renin activity and aldosterone.

### 4.3 Plausible mechanisms

Our research informs potential mechanisms through which SB may influence CVD (Mueller, 2008; Pinto et al., 2023). During acute and prolonged SB, gravity and reduced muscular activity in the legs result in lower extremity blood pooling, which reduces venous return and BP (Pinto et al., 2023). Such alterations can trigger a cascade of cardio‐autonomic and cardio‐metabolic reactions. Baroreceptors sense the reduction in BP and convey the information to the autonomic centre in the brainstem, which suppresses vagal activity, increases the sympathetic outflow (Pinto et al., 2023) and stimulates renin secretion (Mueller, 2008). The latter reaction further activates the RAAS, ultimately resulting in increased BP, which is commonly observed following prolonged SB. Reduced muscle activity during SB can also adversely impact the body's metabolism, leading to impaired glucose, insulin and lipid functions (Pinto et al., 2023). Over time, the cardio‐autonomic and cardio‐metabolic alterations could become chronic maladaptations, leading to CVD (Mueller, 2008; Pinto et al., 2023). Reducing SB, especially prolonged bouts, may prevent the above cascade of adverse cardio‐autonomic and cardio‐metabolic reactions and, potentially, improve cardiovascular health. Our findings that RAAS activity was lower in the intervention versus the control condition support this mechanism. However, the null findings for HRV, glucose, insulin and lipids may indicate that the RESET‐BP intervention may not be adequate with respect to dose (e.g., intensity, duration) or that such mechanisms are not important contributors to the link between SB and CVD.

### 4.4 Strengths

The RESET‐BP trial was conducted with rigour, including blinded assessors, an intervention implemented with high fidelity, and assessments that followed best practices for measuring HRV and cardio‐metabolic biomarkers (Laborde et al., 2017). Furthermore, we quantify the intervention's effects on understudied pathophysiological mechanisms of CVD hypothesized to improve by reducing SB. Combined with the primary study outcomes, our comprehensive study provides empirical data supporting the presence or absence of key pathophysiological pathways that can be used to inform and optimize cardiovascular benefits through SB reduction interventions.

### 4.5 Limitations

The intervention was limited by its short duration (3 months), and behaviour changes were primarily confined to occupational SB, which was mainly relaced by standing. The effects of longer‐term SB reduction, reducing leisure SB, and replacing SB with non‐standing activities are important future directions. Another limitation is that the HRV indices measured did not assess cardio‐sympathetic dysregulation, a key physiological mechanism linked to CVD (Kiyono et al., 2016). Our secondary analysis among the subsample with complete data reduced generalizability and statistical power. Lastly, the RESET‐BP participants had elevated BP that did not require pharmacotherapy and were somewhat active at baseline; individuals who are older, less active or with more advanced CVD risk factors may have realized greater benefits to HRV and cardio‐metabolic biomarkers. Additionally, future research comparing the effects of SB reduction in individuals with and without high blood pressure could further clarify the impact of reducing SB on HRV and cardio‐metabolic biomarkers related to hypertension.

### 4.6 Clinical implications

The current findings indicate that replacing sitting with mostly standing and minimal stepping does not appear to be a sufficient stimulus to improve clinical cardiovascular biomarkers. Adding MVPA and possibly greater amounts of light‐intensity movement may be necessary to achieve health benefits in SB reduction interventions. Still, short‐term reduction in SB through substitution with standing and minimal stepping may suppress RAAS activity in desk workers with elevated, untreated BP. Importantly, heightened RAAS activity over time contributes to CVD, renal diseases and cancer development (Fularski et al., 2024; Hassani et al., 2023; Zhao et al., 2025). Though further randomized controlled trials confirming our findings are important next steps, behavioural modification with SB reduction may be a non‐pharmacological approach to modulate RAAS activity.

### 4.7 Conclusion

We found that SB reduction among desk workers with untreated high BP did not affect resting HRV, fasting glucose, insulin, or lipids over 3 months. In contrast, the trial revealed a previously unexplored, favourable effect on plasma renin and aldosterone. These latter findings encourage further research on how SB influences RAAS as a pathway to CVD risk reduction.

## AUTHOR CONTRIBUTIONS

Abdullah Bandar Alansare, Matthew F. Muldoon, and Bethany Barone Gibbs: Conceptualization. Abdullah Bandar Alansare, Matthew F. Muldoon, and Bethany Barone Gibbs: Methodology. Subashan Perera: Formal analysis. Abdullah Bandar Alansare, Matthew F. Muldoon, Subashan Perera, Kimberly A. Huber, Molly B. Conroy, John M. Jakicic, and Bethany Barone Gibbs: Investigation. Abdullah Bandar Alansare, Matthew F. Muldoon, Subashan Perera, Kimberly A. Huber, Molly B. Conroy, John M. Jakicic, and Bethany Barone Gibbs: Interpretation. Abdullah Bandar Alansare, Matthew F. Muldoon, Subashan Perera, Kimberly A. Huber, Molly B. Conroy, John M. Jakicic, and Bethany Barone Gibbs: Writing—original draft preparation. Abdullah Bandar Alansare, Matthew F. Muldoon, Subashan Perera, Kimberly A. Huber, Molly B. Conroy, John M. Jakicic, and Bethany Barone Gibbs: Writing—review and editing. All authors have read and approved the final version of this manuscript and agree to be accountable for all aspects of the work in ensuring that questions related to the accuracy or integrity of any part of the work are appropriately investigated and resolved. All persons designated as authors qualify for authorship, and all those who qualify for authorship are listed.

## CONFLICT OF INTEREST

John M. Jakicic reports participation as a member volunteer serving as a Vice President for the American College of Sports Medicine, on the Scientific Advisory Board for Wondr Health, Inc. The other authors report no relevant conflicts of interest.

## Supporting information



Supporting Table 1. Baseline characteristics of included versus not included participants for analyses comparing HRV and cardio‐metabolic biomarkers.

## Data Availability

The data are available upon request from the corresponding author.
