# Supplementary material for: Effects of reducing sedentary behaviour on heart rate variability and cardio‐metabolic biomarkers in desk workers with untreated high blood pressure
Source: Exp Physiol. 2026 Apr 2:10.1113/EP093468. Online ahead of print. doi: 10.1113/EP093468 (PMC13394053; doi:10.1113/EP093468)
Supplement: Supplementary file 1 — Supporting Table 1. Baseline characteristics of included versus not included participants for analyses comparing HRV and cardio‐metabolic biomarkers. [file EPH-9999-0-s001.docx]

**Supplemental Table 1. Baseline Characteristics of Included vs. Not Included Participants for Analyses Comparing HRV and Cardio-metabolic Biomarkers**

|  | HRV Analysis | |  | Cardio-metabolic Biomarker Analysis | |  |
| --- | --- | --- | --- | --- | --- | --- |
|  | Included (n=146) Mean ± SD or N (%) | Not Included ^a^ (n=125) Mean ± SD or N (%) | p-value | Included (n=188) Mean ± SD or N (%) | Not Included ^a^ (n=83) Mean ± SD or N (%) | p-value |
| Age (years old) | 44.3 ± 10.8 | 46.3 ± 12.4 | 0.1552 | 45.9 ± 44.2 | 43.7 ± 11.3 | 0.1556 |
| Sex  Female  Male | **74 (50.7)**  **72 (49.3)** | **89 (71.2)**  **36 (28.8)** | **0.0006** | 106 (56.4)  82 (43.6) | 57 (68.7)  26 (31.3) | 0.0568 |
| Ethnicity  Non-Hispanic  Hispanic  Not reported | 140 (95.9)  5 (3.4)  1 (0.7) | 120 (96.0)  5 (4.0)  0 (0.0) | 1.0000 | 179 (95.2)  9 (4.8)  0 (0.0) | 81 (97.6)  1 (1.2)  1 (1.2) | 0.1400 |
| Race  White  Black  Pacific Islander  Asian  Other  Multiracial | **126 (86.3)**  **10 (6.9)**  **1 90.7)**  **3 (2.1)**  **4 (2.7)**  **2 (1.4)** | **100 (80.0)**  **11 (8.8)**  **0 (0.0)**  **12 (9.6)**  **0 (0.0)**  **2 (1.6)** | **0.0182** | 164 (87.2)  10 (5.3)  1 (0.5)  8 (4.3)  2 (1.1)  3 (1.6) | 62 (74.7)  11 (13.3)  0 (0.0)  7 (8.4)  2 (2.4)  1 (1.2) | 0.0730 |
| Resting BP (mmHg)  Systolic  Diastolic | 128.2 ± 8.7  83.1 ± 6.7 | 129.3 ± 8.9  82.5 ± 6.6 | 0.3188  0.4221 | 129.3 ± 9.1  82.7 ± 6.8 | 127.3 ± 7.9  83.1 ± 6.4 | 0.0792  0.6196 |
| BMI (kg/m^2^) | 31.3 ± 6.5 | 30.3 ± 6.6 | 0.1826 | **30.2 ± 6.2** | **32.2 ± 7.1** | **0.0214** |

BMI; body mass index, BP; blood pressure, kg; kilogram, kg/m2; kilogram per meter squared, mmHg; millimeter of mercury, N; number, SD; standard deviation. ^a^ Participants were excluded if missing either baseline or follow-up heart rate variability**.** Missing data occurred due to initiation of HRV testing mid-trial, missed assessments at follow-up, and invalid scans. ^b^ Participants were excluded if missing either baseline or follow-up blood samples. Bold indicates significant difference (p < 0.05).
